# Involuntary Medication, Seclusion, and Restraint in German Psychiatric Hospitals after the Adoption of Legislation in 2013

**DOI:** 10.3389/fpsyt.2015.00153

**Published:** 2015-10-28

**Authors:** Erich Flammer, Tilman Steinert

**Affiliations:** ^1^Clinic for Psychiatry and Psychotherapy I, Centers for Psychiatry Suedwuerttemberg, Versorgungsforschung Weissenau, Ulm University, Ravensburg, Germany

**Keywords:** coercion, seclusion, restraint, physical, forced medication, involuntary treatment

## Abstract

**Background:**

Involuntary medication in psychiatric treatment of inpatients is highly controversial. While laws regulating involuntary medication have been changed in Germany, no data have been available to date on how often involuntary medication is actually applied. Recently, our hospital group introduced specific routine documentation of legal status and application of involuntary medication in the patients’ electronic records, which allows the assessment of the frequency of involuntary medication.

**Method:**

For the year 2014, we extracted aggregated data from the electronic database on age, sex, psychiatric diagnosis, legal status during admission, kind of coercive measure (mechanical restraint, seclusion, and involuntary medication) applied, and the number and duration of seclusion and restraint episodes for seven study sites.

**Results:**

A total of 1,514 (9.6%) of 15,832 admissions were involuntary. At least one coercive measure was applied in 976 (6.2%) admissions. Seclusion was applied in 579 (3.7%) admissions, mechanical restraint was applied in 529 (3.3%) admissions, and involuntary medication was applied in 78 (0.5%) admissions. Two-thirds of involuntary medications were applied in cases of emergency; the remainder was applied after a formal decision by a judge. In 55 (70.5%) of the admissions with involuntary medication, at least one other coercive measure (seclusion, restraint, or both) was applied as well.

**Conclusion:**

Involuntary medication is rarely applied and less frequent than seclusion or mechanical restraint, possibly as a consequence of recent legal restrictions.

## Introduction

The UN Convention on the Rights of Persons with Disabilities (UN-CRPD) (1) was adopted by the United Nations General Assembly in 2006 and has been ratified by 159 states to date (2). Its purpose is “to promote, protect, and ensure full and equal enjoyment of all human rights and fundamental freedoms by all people with disabilities” (1). According to article 1 of the CRPD, it is fully applicable to the situation of people with mental disorders. Article 12 states that “persons with disabilities enjoy legal capacity on an equal basis with others in all aspects of life” (1). The Committee on the Rights of Persons with Disabilities, the body competent to monitor the implementation of the CRPD, makes clear that supported decision-making, involuntary admissions to mental health facilities and forced mental health treatment are inacceptable (3). Although the purpose of the CRPD is unquestioned, there is some concern that its impact might be paradoxical and violate several fundamental rights (4). In its statement to the demands of the Committee, the German government argues that the main problems of people with disabilities concerning legal capacity are not due to laws regulating guardianship themselves but rather to their practical realization (5).

In Germany, coercive interventions in psychiatry are regulated through the laws of guardianship (Betreuungsrecht), which, as federal law, is valid everywhere in the country and in public laws (comparable to mental health acts in other countries), with slightly different regulations in the 16 German federal states (Bundesländer) [“Psych-KG,” “Unterbringungsgesetz” (Law on Compulsory Hospitalization)]. For about a decade, involved stakeholders have expected that it would be necessary to consider involuntary admission and involuntary treatment separately in law texts as some European countries and the United States have done since 1980. This happened in 2011 when the Constitutional Court (Bundesverfassungsgericht) ruled on two decisions concerning involuntary medication in forensic psychiatry, which had a huge impact on clinical practice. In both cases, the Constitutional Court decided the existing laws regulating involuntary medication were unconstitutional and, thus, were no longer valid. The Constitutional Court determined that law texts should clarify that involuntary medication could be applied only for people without capacity to consent, and only as a last measure if all other approaches had failed, particularly after intensive attempts to convince the patient. Moreover, this should be allowed only after a separate court decision, and taking into account the review of an independent expert. Though this decision was primarily related to forensic psychiatry, other supreme courts (Bundesgerichtshof) adopted this view and extended it to all kinds of involuntary treatment, including that given to general psychiatric patients. Consequently, involuntary medication was no longer a legitimate means of psychiatric treatment, except for acute emergencies, until new legislations fulfilling the Constitutional Court’s requirements were adopted in 2013. This referred to guardianship law as well.

These changes in legislation and jurisdiction have caused intense discussions among psychiatrists, patients’ organizations, ethical boards, jurists, and political stakeholders. Although many statements have been published, such as ones from the German Association for Psychiatry and Psychotherapy and the ethical committee of the Federal Chamber of Physicians, among others, no data are currently available to show how often involuntary medication really happens. Due to the lack of clear definition and defined legal procedures, no statistics had been collected. The same applies to most other countries. In 2013, our hospital group introduced specific routine documentation of legal status and application of involuntary medication in the patients’ electronic records. Thus, data on the use of coercive medication in psychiatric hospitals in Germany are available for the first time, subdivided according to the respective legal basis. Furthermore, because reliable data on freedom-restricting coercive measures, such as seclusion and mechanical restraint is also available, according to a thoroughly implemented routine documentation, the frequency of coercive medication can be related to the frequency of freedom-restrictive interventions.

## Materials and Methods

### Study Sites

The Centers for Psychiatry Suedwuerttemberg are a psychiatric organization providing inpatient and outpatient mental health care and serving a catchment area of about 1.2 million inhabitants in southwest Germany. The seven psychiatric inpatient units are four psychiatric hospitals and three psychiatric departments of general hospitals with a total of about 1,100 beds and 15,000 admissions per year. All of them are responsible for a defined catchment area, including all involuntary admissions. The number of beds amounts to ~93/100,000 inhabitants. Although no other psychiatric inpatient units are available in the respective areas, alternatives to inpatient treatment exist with day clinics, outpatient units and highly developed community services, including home treatment.

### Data Sources

The centers operate an extensive electronic database of routinely collected clinical information from basic patient documentation [Basisdokumentation (BADO)]. The database also contains information on the use of seclusion and restraint, including exact data on the duration of each measure and is collected according to clear definitions (6). These data can be considered highly accurate due to the legal obligations of documentation. In general, the patient characteristics of the BADO documented in the electronic charts is sufficiently valid and reliable (7).

For the year 2014, we extracted the following aggregated data from all hospital admissions in the seven hospitals that existed in the electronic database: age, sex, psychiatric diagnosis according to ICD-10, legal status during admission (whether voluntary or detained according to guardianship legislation or whether detained, according to mental health legislation), kind of coercive measure (if it occurred: mechanical restraint, seclusion, or involuntary medication as either an emergency measure or involuntary medication after judge’s decision), number of coercive measures per admission, and the duration of mechanical restraint or seclusion. Thus, all presented data refer to admissions, not to patients; the number of patients is lower than the number of admissions due to readmissions.

### Definitions

Admissions were classified as “involuntary” if patients stayed involuntarily in the hospital at any time. This included all cases with court decisions on involuntary commitment but also those in which patients changed their mind after a short time and subsequently stayed on a voluntary basis before application to a court.

Seclusion is defined as bringing the patient into a locked room where he or she is alone and able to move freely but unable to leave due to a locked door (8). During seclusion, patients are observed through a window in the door of the seclusion room or by video monitoring.

Mechanical restraint refers to the use of belts to fix the patient to the bed (8). According to internal hospital guidelines, patients have to be constantly and personally monitored during mechanical restraint.

Involuntary medication is defined according to an agreement of the German working groups on violence and coercion as any medication, oral or per injection, against the patient’s explicit or implicit will (9). In the ambiguous area of psychological pressure, medication is classified as “involuntary,” if patients have no other choice than being subjected to a different kind of coercive measure if they would refuse medication. Two types of involuntary medication can be distinguished, according to the current legal situation. Involuntary medication can be applied in case of acute emergency if required to prevent serious harm (in this case, the physician remains free of punishment according to the penalty law, although no special legal basis exists). The other case for involuntary medication is after a judge’s decision based on an independent expert’s review, which can occur as a matter of guardianship law as well as public law.

### Ethical Considerations

Privacy of patients and confidentially of personal information was ensured by using solely aggregated data (e.g., absolute numbers, means, and proportions) but not identifiable individual data. As retrospective, aggregated data from electronic databases was used, ethical committee approval was not considered necessary according to German regulations.

## Results

The sample consisted of 15,832 admissions (7,656 women, 48.4%). The mean age was 45.9 years (SD = 18.8), for women it was 47.9 (SD = 19.2) and for men 43.7 (SD = 18.1). The admissions totaled over 10,181 patients (4,995 women, 49.1%). Of the 15,832 admissions, 1,514 (9.6%) of them were involuntary: 1,026 (6.5%) of these were admitted according to mental health legislation and 488 (3.1%) were admitted according to guardianship legislation. Table 1 shows the main diagnoses (ICD-10) and the length of stay according to the legal status of admission.

**Table 1:** **Main diagnoses (ICD-10) and length of stay according to legal status**.

Main diagnosis (ICD-10)	Legal status^a^			Total (*N* = 15,832)
	Voluntary (*n* = 14,318)	Detained according to mental health legislation (*n* = 1,026)	Detained according to guardianship legislation (*n* = 488)	
F0/G3	965 (6.7%)	136 (13.3%)	146 (29.9%)	1,247 (7.9%)
F1	4,544 (31.7%)	282 (27.5%)	60 (12.3%)	4,886 (30.9%)
F2	2,050 (14.3%)	383 (37.3%)	194 (39.8%)	2,627 (16.6%)
F3	3,724 (26.0%)	96 (9.4%)	25 (5.1%)	3,845 (24.3%)
F4	1,895 (13.2%)	77 (7.5%)	32 (6.6%)	2,004 (12.7%)
F5	52 (0.4%)	0 (0.0%)	0 (0.0%)	52 (0.3%)
F6	795 (5.6%)	51 (5.0%)	18 (3.7%)	864 (5.5%)
F9	293 (2.0%)	1 (0.1%)	13 (2.7%)	307 (1.9%)
Length of stay (days; mean, SD)	26.7 (26.9)	30.5 (40.9)	47.4 (53.1)	27.6 (29.3)

*^a^All percentages reflect the proportion of the column total*.

At least one coercive measure was applied in 976 admissions (6.2%) of which 424 were women (43.4%). The number and the duration of each kind of coercive intervention and the percentage of affected admissions according to legal status are presented in Table 2.

**Table 2:** **Affected cases, number, and duration of coercive measures according to legal status**.

Legal status	Coercive measure^a^				Total^b^
	Seclusion	Mechanical restraint	Involuntary medication as an emergency measure	Involuntary medication after judge’s decision	
Voluntary (*n* = 14,318)	263 (1.8%)	228 (1.6%)	12 (0.1%)	0 (0.0%)	455 (3.2%)
Detained according to mental health legislation (*n* = 1,026)	187 (18.2%)	220 (21.4%)	31 (3.0%)	17 (1.7%)	347 (33.8%)
Detained according to guardianship legislation (*n* = 488)	129 (26.4%)	81 (16.6%)	17 (3.5%)	12 (2.5%)	174 (35.7%)
Total (*N* = 15,832)	579 (3.7%)	529 (3.3%)	60 (0.4%)	29 (0.2%)	976 (6.2%)
Number of coercive measures	3,016	1,962			5,125
Duration (h; mean, SD)	5.5 (7.9)	8.1 (7.2)			6.5 (7.7)

*^a^All percentages reflect the proportion of the row total*.

*^b^Admissions who suffered more than one coercive measure are only totaled once*.

The most frequent main diagnosis among those admissions was schizophrenia, schizotypal, or delusional disorder (ICD-10 F2, 393 admissions, 40.3%). The number of affected cases for each main diagnosis is presented in Table 3. Seclusion was applied in 579 admissions (3.7%) and 529 admissions (3.3%) received mechanical restraint. The total number of seclusions was 3,016 (5.2 measures per affected admission), and the total number of mechanical restraints was 1,962 (3.7 measures per affected admission). The mean duration of seclusion was 5.5 h (SD = 7.9), and the mean duration of mechanical restraint was 8.1 h (SD = 7.2). Involuntary medication was applied in 78 admissions (0.5%). Among the admissions with involuntary medication the main diagnosis of schizophrenia, schizotypal, or delusional disorder (ICD-10 F2) was most frequent (57 admissions, 73.1%). In 55 (70.5%) of those admissions affected by involuntary medication at least one other coercive measure (seclusion, restraint, or both) was applied as well.

**Table 3:** **Cases with coercive measures according to main diagnosis**.

Main diagnosis (ICD-10)	Coercive measure^a^				Total^b^
	Seclusion	Mechanical restraint	Involuntary medication as an emergency measure	Involuntary medication after judge’s decision	
F0/G3 (*n* = 1,247)	55 (4.4%)	146 (11.7%)	13 (1.0%)	1 (0.1%)	196 (15.7%)
F1 (*n* = 4,886)	77 (1.6%)	95 (1.9%)	2 (0.0%)	1 (0.0%)	163 (3.3%)
F2 (*n* = 2,627)	279 (10.6%)	187 (7.1%)	40 (1.5%)	26 (1.0%)	393 (15.0%)
F3 (*n* = 3,845)	43 (1.1%)	36 (0.9%)	2 (0.1%)	1 (0.0%)	66 (1.7%)
F4 (*n* = 2,004)	48 (2.4%)	15 (0.7%)	1 (0.0%)	0 (0.0%)	58 (2.9%)
F5 (*n* = 52)	1 (1.9%)	1 (1.9%)	0 (0.0%)	0 (0.0%)	1 (1.9%)
F6 (*n* = 864)	65 (7.5%)	46 (5.3%)	2 (0.2%)	0 (0.0%)	87 10.1(%)
F9 (*n* = 307)	11 (3.6%)	3 (1.0%)	0 (0.0%)	0 (0.0%)	12 (3.9%)

*^a^All percentages reflect the proportion of the row total*.

*^b^Admissions who suffered more than one coercive measure are only totaled once*.

## Discussion

This is the first data on the use of coercive medication in Germany since the introduction of the legislation increased the threshold for the application of such measures in 2013. These results from seven psychiatric departments and hospitals in Germany show that 6% of over 15,000 admissions in 2014 were subjected to some kind of coercive measures. Involuntary medication was applied in less than a tenth (0.5%) of those cases. Two-thirds of coercive medications were applied in cases of emergency; only 0.2% of admissions received involuntary medication after a judge’s decision. Because data on the previous practice are only scarcely available, it is difficult to say whether the percentage of exposed patients has decreased after the introduction of the legislation. Estimations of percentage of affected patients vary between 0.4 and 5.6% (10–12). According to these data, there is some uncertain evidence that the use of involuntary medication has considerably decreased after the introduction of the legislation, which had been intended. However, a different question is whether this is good news for patients. In a previous analysis, we found a doubling of the numbers of seclusion and restraint and of patient assaults on staff during the temporary lack of admissibility of involuntary medication due to a gap in legislation for some months between 2012 and 2013 (13). While the threshold for involuntary medication has been increased in Germany in recent years, the Netherlands went the opposite way after realizing that a restriction of medication had led to a dramatic increase in the use of seclusion and in patient assaults (14, 15). Recently, evidence has become available that those hospitals using less medication use more seclusion and vice versa (16). However, data after a policy change in New York concerning unscheduled psychotropic medication for agitation in an acute psychiatric inpatient setting indicate that a reduction in medication does not necessarily derogate safety (17). While PRN (*pro re nata*, i.e., “as needed”) medication decreased significantly after a mandated change in utilization, there was no significant difference in rates of assault or seclusion 7.5 months before and after that policy change. In the course of an overlapping hospital project that aimed to reduce the use of restraints, the restraint rate significantly decreased over the period of review.

So, further developments should be observed carefully, which may possibly be helped by the introduction of a statewide register for coercive measures, as required by the new Mental Health Act (2015) in the state of Baden-Württemberg (18).

The most important limitation of the presented data is that it is grounded on a very limited number of hospitals. It remains unclear to which extent these data are representative of psychiatric hospitalization practices in Germany as a whole. Another concern might be that involuntary medication by definition did not include subtle forms of pressure on a patient to take medication which can be experienced as “coercive” without fulfilling formal criteria of coercion according to the definition applied (9). So from patient’s perspective, the finding that only one in 200 patients received involuntary medication could be an underestimation of the real problem.

## Conflict of Interest Statement

The authors declare that the research was conducted in the absence of any commercial or financial relationships that could be construed as a potential conflict of interest.
